# Pharmacological Management of Mild Cognitive Impairment: From Symptomatic Treatment to Disease Modification—A Narrative Review

**DOI:** 10.3390/neurosci7010002

**Published:** 2025-12-26

**Authors:** Andrei Gabriel Mangalagiu, Bogdan Mircea Petrescu, Sorin Riga, Octavian Vasiliu

**Affiliations:** 1 Discipline of Psychiatry II, Department of Clinical Neurosciences, Carol Davila University of Medicine and Pharmacy, 050474 Bucharest, Romania; bogdan.petrescu@umfcd.ro; 2 Department of Psychiatry, Dr. Carol Davila University Emergency Central Military Hospital, 010816 Bucharest, Romania; 3 Romanian Academy of Medical Sciences, 030171 Bucharest, Romania; d_s_riga@yahoo.com

**Keywords:** mild cognitive impairment, major neurocognitive disorder, mild neurocognitive disorder, donanemab, lecanemab, citicoline, *Ginkgo biloba*, racetams, cholinesterase inhibitors, memantine

## Abstract

Mild cognitive impairment (MCI) is a nosological entity that requires special attention from a therapeutic perspective, because annual conversion rates to dementia of 5–15% in these cases are considered typical. This narrative review aimed to identify available data supporting the efficacy and tolerability of various pharmacological therapeutic interventions by searching PubMed/MEDLINE, the Cochrane Database of Systematic Reviews, and the Web of Science (WoS) Core Collection for primary and secondary reports published over the last 25 years on the pharmacological treatment of MCI. The retrieved interventions were distributed in five large categories: (1) conventional cognitive enhancers; (2) disease-modifying therapeutic interventions; (3) strategies mitigating vascular risk and management of concomitant medications; (4) adjuvant and nootropic formulations; (5) case management of non-cognitive symptoms in MCI. The most broadly applicable pharmacological strategies in MCI include systematic deprescribing and optimisation of concomitant therapies, reducing anticholinergic and sedative load, avoiding iatrogenic hypoglycaemia and excessive blood pressure lowering, and careful, individualised treatment of vascular risk factors. Based on the randomised controlled trials, meta-analyses, and contemporary guidelines, a pragmatic pharmacological approach to MCI is suggested. Further trials with better design are urgently needed to document the efficacy and safety of pharmacological interventions in patients diagnosed with MCI.

## 1. Introduction

Neurocognitive disorders are an extremely heterogeneous category in clinical psychiatry, encompassing both acute (delirium) and chronic pathologies (dementias, or major and minor neurocognitive disorders, depending on the nosological system used) [1,2,3,4,5]. Several important changes emerged from the ICD-10/DSM-IV TR paradigm to the ICD-11/DSM-5/5 TR perspective on the neurocognitive disorders: (1) a more etiology-based and neuropsychologically grounded framework is visible in the more recent nosological systems, leading to the disappearance of amnestic disorders from this chapter; (2) avoidance of stigmatisation and negative connotations related to the term “dementia” led to its replacement with the more neutral concept of “neurocognitive disorder”; (3) severity levels were introduced by the newest classifications; (4) cognitive domains are better defined in the latest editions; (5) a clear accent is placed on the ability to function independently in DSM-5 TR, as the primary criterion to differentiate neurocognitive disorders [1,2,3,4,5].

In the same analytical line, mild cognitive impairment (MCI), a construct loosely described in ICD-10 (“mild cognitive disorder”) and largely overlapping with mild neurocognitive disorder (mNCD) in DSM-5 TR and ICD-11, refers to objectively measurable cognitive decline occurring in the context of essentially preserved basic functional independence [1,6,7,8,9]. It is typically conceptualised as an intermediate stage between normal ageing and major neurocognitive disorders (MNCDs) [1,2,6,7,8]. Clinically, MCI is characterised by a modest decline in one or more cognitive domains, reported by the patient, an informant, or a clinician and confirmed through neuropsychological assessment, in the absence of substantial impairment in basic activities of daily living [1].

Over the past two decades, our understanding of MCI has changed considerably. Petersen’s original, predominantly amnestic formulation framed it as isolated episodic memory impairment, conceived as a prodromal stage of Alzheimer’s disease (AD) [6]. Later consensus reports went further and broadened the construct, rendering MCI a highly heterogeneous nosological entity [7,10]. The current framework distinguishes four main clinical subtypesamn—estic single-domain (aMCI-SD), amnestic multi-domain (aMCI-MD), non-amnestic single-domain (naMCI-SD), and non-amnestic multi-domain (naMCI-MD) [7,10]. The first one, aMCI-SD, typically presents with isolated episodic memory impairment and relatively preserved performance in other cognitive domains and is often prodromal to AD, with annual conversion rates around 10–15% in memory-clinic cohorts [10,11,12]. The second subtype, aMCI-MD, involves episodic memory plus additional domains (for example, executive function or language) and carries the highest risk of progression to AD or mixed dementia, with annual conversion rates of roughly 20% [10,11,12]. The third subcategory, naMCI-SD, is characterised by isolated non-mnestic deficits (such as language or visuospatial skills) and frequently precedes non-AD dementias, including primary progressive aphasia, dementia with Lewy bodies (DLB), or vascular dementia [10,11]. The last one, naMCI-MD, affects at least two non-mnestic domains and is commonly associated with vascular or mixed pathologies; its annual conversion risk is lower than in aMCI-MD but still higher than that of cognitively normal peers [10,11,12].

Conversion risk also differs markedly between settings. In specialist memory clinics, where the pre-test probability of underlying neurodegenerative pathology is high, annual conversion rates of 10–15% are typical, whereas in community-based screening studies, which include milder and more benign cases, rates are closer to 5–10% per year [12,13]. Stability and “reversion” to normal cognition are frequent as well: approximately half of individuals with MCI remain stable over 3–5 years, and 9–28% revert to normal on follow-up, particularly in population cohorts and single-domain phenotypes [13,14].

Longitudinal studies have identified multiple predictors of progression to dementia, including lower baseline memory performance, involvement of multiple cognitive domains, positive AD biomarkers (amyloid and tau), APOE ε4 genotype—especially ε4/ε4—neuropsychiatric features such as depression, apathy, and anosognosia, imaging markers such as hippocampal atrophy or temporoparietal hypometabolism, low cognitive reserve, and uncontrolled vascular risk factors [10,11,12,15]. Taken together, these data support a stratified risk model rather than a uniform view of MCI as “early AD”. From a pharmacological standpoint, it is therefore more rational to consider drug treatment in high-risk phenotypes (for example, aMCI-MD with positive AD biomarkers) than in low-risk, potentially reversible forms such as non-amnestic MCI with predominant vascular or comorbid psychiatric disorders.

More recently, the introduction of research criteria for “MCI due to AD” and the “A/T/N” framework has shifted the focus from a purely clinical syndrome to a biologically anchored disease stage, in which amyloid, τ, and neurodegeneration biomarkers play a central role in defining and classifying MCI [9].

This nosological shift has direct therapeutic implications. Amnestic multi-domain MCI with positive AD biomarkers and APOE ε4 homozygosity carries a very high short-term risk of conversion to AD dementia, whereas non-amnestic single-domain MCI is more likely to remain stable or to evolve toward non-Alzheimer dementias [10,11,12,15]. In contrast, roughly half of individuals with MCI show clinical stability over 3–5 years, and a substantial proportion revert to apparently normal performance, particularly in population-based cohorts [13,14]. Against this background, it is difficult to argue that a single pharmacological strategy applied across all MCI phenotypes would yield consistent benefit.

Also, when discussing the clinical criteria and evolution of MCI, the differential diagnosis is of major importance to avoid overestimating this condition and to create unnecessary case management complications [2,16]. Chronic psychoses with deteriorative cognitive trajectory, organic personality disorders with cognitive impairment, delirium, well-defined from an etiologically perspective mNCDS and MNCDs, old-age depressive disorder with mnestic deterioration (“pseudodementia”), anticholinergic, benzodiazepine, or antihistamine drugs intoxication, substance use disorders (including Wernicke–Korsakoff syndrome), various organic conditions with reversible impact on cognitive functions (e.g., obstructive sleep apnoa, hypothyroidism), and normal aging are but a few of MCI differentials [2,16,17].

In parallel, a wide range of pharmacological approaches has been tested in MCI, often with modest or inconsistent benefits: classical cognitive enhancers (cholinesterase inhibitors- ChEI and memantine), neurotrophic agents (Cerebrolysin^®^), cholinergic precursors (citicoline, choline alphoscerate), herbal extracts (standardised *Ginkgo biloba* EGb 761^®^), various vitamins and nutraceuticals, and, more recently, monoclonal antibodies targeting amyloid-β in biomarker-defined early AD. At the same time, accumulating evidence suggests that some commonly prescribed medications, particularly highly active anticholinergics and high-potency benzodiazepines, together with uncontrolled vascular risk factors, can themselves accelerate cognitive decline.

Most major guidelines converge toward a cautious stance: no pharmacological intervention can currently be recommended as standard of care for generic MCI, and management remains primarily non-pharmacological, coupled with optimisation of comorbidities and systematic deprescribing of potentially harmful medications [18,19,20,21]. This stance is stated explicitly in the AAN guideline update (2018) [18] and is consistent with the WHO risk-reduction guideline (2019) [20]. The development and conditional approval of anti-β-amyloid monoclonal antibodies for biomarker-confirmed early AD have revived the discussion on disease-modifying treatment in this stage. However, based on currently available evidence, their use remains limited to highly selected patients (e.g., after the confirmation of amyloid plaques, structured tau staging, and genetic risk assessment) [22,23,24,25,26].

In this narrative review, based on the need to better understand the level of evidence for pharmacological approaches in patients diagnosed with MCI, we synthesise and critically appraise the available data in the literature. A broad perspective on the concept of pharmacological treatment was adopted, ranging from classical cognitive enhancers and disease-modifying anti-β-amyloid therapies in early AD to indirect psychopharmacological approaches through vascular risk control and adjustment of concomitant medications, as well as adjuvant nootropic and nutraceutical interventions.

## 2. Materials and Methods

We conducted a narrative, non-systematic review of pharmacological strategies in mild cognitive impairment (MCI), focusing on adult patients with MCI or mNCD as defined by contemporary diagnostic criteria, with particular emphasis on agents targeting cognitive symptoms or aiming to modify the disease.

We performed a targeted literature search in PubMed/MEDLINE, the Cochrane Database of Systematic Reviews, and the Web of Science (WoS) Core Collection from 1 January 2000 to 30 September 2025 and supplemented it by hand-searching the reference lists of key papers. To complement this search, the official websites of major clinical practice guideline and consensus-issuing organizations (e.g., World Health Organization [WHO], National Institute for Health and Care Excellence [NICE], American Psychiatric Association [APA]) were examined to identify the most relevant recommendations regarding MCI pharmacological treatment.

Our PubMed search combined terms using Boolean operators and included variations of the following: “mild cognitive impairment”, “mild neurocognitive disorder”, “cholinesterase inhibitor”, “donepezil”, “rivastigmine”, “galantamine”, “memantine”, “Cerebrolysin”, “citicoline”, “choline alphoscerate”, “*Ginkgo biloba*”, “EGb 761”, “vitamin B”, “vitamin D”, “vitamin E”, “piracetam”, “nootropics”, “monoclonal antibody”, “lecanemab”, “donanemab”, “aducanumab”, “anticholinergic burden”, “benzodiazepine”, “hypertension”, “statins”, and “neuropsychiatric symptoms”. Only articles published in English and involving human participants were considered.

Titles and abstracts were screened for relevance to one or more of the following domains: (a) pharmacological agents evaluated in MCI or very early Alzheimer’s disease, particularly in randomised controlled trials (RCTs) or their meta-analyses; (b) systematic reviews and meta-analyses addressing cognitive enhancers, nootropics, vitamins, or vascular/metabolic interventions in older adults with cognitive impairment; (c) major clinical practice guidelines or consensus documents on the diagnosis and management of MCI and dementia; and (d) observational studies assessing associations between concomitant medications, vascular risk control, and cognitive outcomes. We prioritised drug classes that either had at least one RCT in an MCI or “early AD” population or were explicitly discussed in influential guidelines or consensus statements (for example, cholinesterase inhibitors, memantine, anti-amyloid monoclonal antibodies, EGb 761^®^, citicoline, choline alphoscerate, Cerebrolysin, and vitamin regimens).

Preclinical and purely mechanistic work, case reports and small case series, paediatric samples, and trials centred on non-cognitive indications unrelated to MCI or dementia were excluded. When doubt existed about eligibility—for example, in early AD samples partially overlapping with biomarker-defined “MCI due to AD”—we retained studies if the population clearly included individuals at the MCI/very mild dementia boundary and the results were clinically applicable to that stage.

### Study Prioritisation and Qualitative Appraisal

Because this is a narrative (non-systematic) review, we did not apply a formal risk-of-bias instrument. Instead, when multiple trials or meta-analyses were available within the same drug class, we prioritised evidence using a pragmatic hierarchy focused on interpretability and clinical relevance: (i) randomised controlled trials and their pooled syntheses were weighted over observational data; (ii) among meta-analyses, we favoured those with clearly defined inclusion criteria, contemporary search windows, and consistent prespecified outcomes; (iii) “key” studies were those that were pivotal to guideline and practice discussions (e.g., large multicentre RCTs or landmark phase 3 programmes); and (iv) we considered methodological features that materially affect conclusions (randomisation/blinding, attrition, outcome multiplicity, and external validity to typical MCI care). Where signals relied on subgroup analyses, short follow-up, or small regional cohorts, we framed conclusions as provisional and adjunctive rather than definitive.

## 3. Results

Data retrieved were distributed in five large categories: (1) conventional cognitive enhancers; (2) disease-modifying therapeutic interventions; (3) mitigating risk factors for MCI and conversion from MCI to dementia; (4) adjuvant and nootropic formulations; (5) case management of non-cognitive symptoms in MCI.

### 3.1. Conventional Cognitive Enhancers: Cholinesterase Inhibitors and Memantine

#### 3.1.1. Donepezil

Donepezil is the most extensively investigated ChEI in patients presenting with MCI. The Alzheimer’s Disease Cooperative Study on MCI (ADCS-MCI) trial randomised 769 individuals with amnestic MCI to donepezil 10 mg/day, vitamin E 2000 IU/day, or placebo for three years, with conversion to probable AD as the primary outcome [27]. Over three years, the cumulative probability of progression to Alzheimer’s disease did not differ significantly between the donepezil and placebo groups (hazard ratio 0.80; 95% CI 0.57–1.13) [27]. A reduction in conversion risk was observed during the first 12–18 months, particularly among APOE ε4 carriers, but this benefit was not sustained with longer follow-up and was based on subgroup analyses [27]. Adverse events, notably gastrointestinal symptoms and insomnia, were more frequent with donepezil and led to higher discontinuation rates [27].

Subsequent trials have produced broadly concordant results. A 48-week randomised study in 821 participants with MCI reported modest improvements on some cognitive measures with donepezil but no clinically meaningful benefit in global functioning [28]. Meta-analyses reinforce this pattern. The Cochrane review of ChEIs in MCI found no robust effect of donepezil on cognition or progression to dementia, alongside a clear excess of adverse events [29]. A later meta-analysis by Matsunaga et al. (2019) reported small effect sizes on Mini-Mental State Examination (MMSE) at 10 mg/day but no impact on the Alzheimer’s Disease Assessment Scale–Cognitive Subscale (ADAS-Cog) and substantial heterogeneity across studies, while Zhang et al. (2022) found improvements on MMSE and MoCA but again no effect on ADAS-Cog and overall low quality of evidence [30,31].

Taken together, the data indicate that donepezil yields, at best, small and transient changes in cognitive test scores without durable prevention of dementia, while imposing a non-trivial burden of adverse effects. In MCI, short-term changes on repeated cognitive scales should be interpreted cautiously, as test–retest variability, including practice effects, and outcome multiplicity may inflate apparent improvements when functional endpoints do not move in parallel.

#### 3.1.2. Rivastigmine

Rivastigmine has also been evaluated in MCI. The InDDEx trial enrolled 1018 participants with MCI and followed them for up to four years [32]. Rivastigmine did not significantly delay conversion to AD compared with placebo (17.3% vs. 21.4%; hazard ratio 0.85; *p* = 0.22), nor did it provide clear advantages on global cognitive outcomes. Adverse events were very common—around 95% of participants experienced at least one event, most often gastrointestinal symptoms—although the proportion of serious events was similar in the rivastigmine and placebo groups [32].

Smaller studies in vascular cognitive impairment have hinted at possible benefits of rivastigmine on executive function, but these findings are too limited and context-specific to allow confident generalisation to unselected MCI populations [33]. Overall, available data do not demonstrate a consistent benefit on global or functional outcomes in MCI, whereas adverse effects—predominantly gastrointestinal—remain frequent.

#### 3.1.3. Galantamine

Galantamine has been examined in multiple trials, including the GAL-INT programme, which enrolled around 1900 individuals with MCI and followed them over two years [34]. No significant benefits were seen on the Alzheimer’s Disease Assessment Scale–Cognitive Subscale (ADAS-Cog) or on the Clinical Dementia Rating–Sum of Boxes (CDR-SB). The galantamine arm showed a high discontinuation rate, predominantly driven by gastrointestinal adverse events, and some analyses raised concerns about a potential increase in mortality, although this signal has not been consistently reproduced across studies [34].

A recent Cochrane review concluded that galantamine has minimal or no impact on cognitive performance or activities of daily living in MCI and does not clearly lower the risk of progression to dementia, while clearly increasing the likelihood of gastrointestinal side effects and treatment withdrawal [35].

#### 3.1.4. Memantine

Memantine, an N-methyl-D-aspartate (NMDA) receptor antagonist approved for moderate-to-severe AD, has been much less extensively studied in MCI. A small 48-week pilot randomised controlled trial in 25 patients with amnestic MCI reported improvements in MMSE scores and semantic memory with memantine compared with control [36]. However, the sample size was very limited and the study was underpowered for clinically meaningful endpoints such as conversion to dementia.

Syntheses of memantine trials in early AD and MCI converge on the view that there is no convincing evidence of efficacy in the MCI stage and only modest data even in mild AD [37]. Although memantine is generally better tolerated than ChEIs, with dizziness, headache, and constipation as the most frequently reported adverse events, available MCI-stage data remain limited and underpowered for clinically anchored endpoints (e.g., sustained functional change or conversion).

Overall, randomised controlled trials and meta-analyses indicate that standard symptomatic treatments for Alzheimer’s disease—ChEIs and memantine—produce only small, often short-lived improvements in cognitive test scores, do not demonstrate a convincing long-term reduction in progression from MCI to dementia, and are consistently associated with higher rates of adverse events, particularly gastrointestinal symptoms and treatment discontinuations [27,28,29,30,31,32,34,35]. In line with these findings, the AAN practice guideline update (2018) states that clinicians may choose not to offer cholinesterase inhibitors in MCI and, if they are offered, the lack of evidence and off-label status should be discussed [18,19,20,21]; memantine likewise lacks convincing evidence for routine use in MCI [36,37]. The possibility of a short, closely monitored therapeutic trial in highly selected cases (e.g., progressive, biomarker-positive amnestic presentations) is addressed in the Discussion and practical algorithm.

### 3.2. Disease-Modifying Therapeutic Interventions in MCI Due to Alzheimer’s Disease

It is important to emphasise that clinical MCI is a heterogeneous syndrome, whereas biomarker-confirmed “MCI due to AD” is an aetiologically defined research construct within that broader clinical spectrum. In this review, disease-modifying anti-amyloid therapies are discussed only in relation to biomarker-confirmed early AD presentations (MCI or very mild dementia consistent with AD) and should not be extrapolated to biomarker-undefined or potentially reversible MCI phenotypes. Broadening AD-specific therapies to unstratified MCI risks diagnostic drift and overmedicalisation, particularly when short-term fluctuations, comorbid psychiatric conditions, or medication effects can mimic “progressive” cognitive symptoms.

Monoclonal antibodies directed against aggregated amyloid-β species (aducanumab, lecanemab, donanemab) have been shown to reduce cerebral amyloid load and to slow clinical decline modestly in the early symptomatic stages of Alzheimer’s disease [38,39,40,41,42,43]. Their potential relevance to MCI is confined to the subgroup meeting research criteria for “MCI due to AD”, in whom amyloid positivity has been demonstrated by positron emission tomography or cerebrospinal fluid biomarkers.

In the CLARITY-AD trial, lecanemab was evaluated in 1795 individuals with early AD (MCI or mild dementia) and confirmed amyloid pathology [38]. Over 18 months of treatment, lecanemab produced approximately a 27% relative slowing of decline on CDR-SB compared with placebo, with concordant benefits across several secondary cognitive and functional measures [38]. In absolute terms, the mean change in CDR-SB at 18 months was 1.21 points in the lecanemab group versus 1.66 points with placebo (difference −0.45 points; 95% CI −0.67 to −0.23) [38]. Although statistically significant, the absolute separation on CDR-SB remains modest and should be interpreted alongside functional concordance, monitoring requirements and treatment burden. Amyloid-related imaging abnormalities, including vasogenic oedema or effusion (ARIA-E) and microhaemorrhages or superficial siderosis (ARIA-H), occurred in about 20–30% of treated participants, necessitating structured MRI surveillance throughout therapy [38].

Donanemab, tested in the TRAILBLAZER-ALZ 2 study in early symptomatic AD, similarly reduced amyloid burden and achieved roughly 30–35% slowing of clinical decline, with the clearest effect in patients with intermediate tau burden on positron emission tomography [39]. In that population, the least-squares mean change in CDR-SB at 76 weeks was modestly lower with donanemab than with placebo, consistent with a small absolute difference despite the relative slowing of decline [39]. As with lecanemab, ARIA events were common and mandated intensive imaging-based monitoring and careful selection of candidates [39]. Also, positive results were seen in the TRAILBLAZER-ALZ 4 study (*N* = 148 participants diagnosed with early, symptomatic AD), where amyloid plaque clearance was achieved in 38%, 70%, and 76.8% after 6, 12, and 18 months, respectively, of treatment with donanemab vs. 1.6%, 24.6%, and 43.1%, respectively, of treatment with aducanumab [43]. In this lastly mentioned trial, ARIA-E occurred in 24 vs. 34.8% in patients treated with donanemab vs. aducanumab [43].

Aducanumab, the first anti-amyloid antibody to receive regulatory approval, yielded discordant phase 3 results: one trial (EMERGE) reported a positive effect on CDR-SB, whereas the companion study (ENGAGE) was negative [40]. Against a backdrop of methodological controversy and implementation challenges, the manufacturer has subsequently halted further development and withdrawn aducanumab from the market, so it no longer constitutes a realistic therapeutic option in routine clinical practice [40].

Current regulatory frameworks limit the use of lecanemab and donanemab to patients who present with a clinical syndrome of MCI or mild dementia consistent with AD, have biomarker-confirmed amyloid pathology (and, in some protocols, tau staging), and show acceptable cerebrovascular risk and brain MRI profiles [38,39,40]. Even within this highly selected population, the absolute magnitude of benefit is modest, whereas the treatment burden—intravenous or frequent subcutaneous administration, repeated MRI scans, and the risk of ARIA—is substantial. Uncertainties regarding cost-effectiveness further constrain implementation; for example, recent guidance (2024–2025) from the UK National Institute for Health and Care Excellence (NICE) does not support routine National Health Service use of lecanemab or donanemab despite evidence of efficacy [44,45,46].

Accordingly, contemporary guidelines restrict these agents to specialised centres, recommend extensive shared decision-making and explicit discussion of risks and uncertainties, and do not endorse their use in unselected MCI. For heterogeneous, biomarker-undefined MCI, available evidence does not support extrapolation of these agents beyond biomarker-confirmed early AD presentations [18,21,38,39,40,44,45,46].

### 3.3. Addressing Vascular Risk and Concomitant Medications

#### 3.3.1. Management of Vascular Risk Factors

Vascular pathology is a major driver of cognitive decline and frequently co-exists with neurodegenerative processes. Hypertension, diabetes, dyslipidaemia, and atrial fibrillation are all associated with an increased risk of MCI and dementia in longitudinal cohorts [12,13,47]. Early work from antihypertensive trials, including the Systolic Hypertension in Europe (Syst-Eur) study, suggested that treatment of isolated systolic hypertension in older adults may reduce the incidence of dementia compared with placebo [47]. Subsequent studies and guideline recommendations have broadly supported the view that adequate blood pressure control lowers the likelihood of cognitive decline and dementia, although the optimal blood pressure targets in very old or frail individuals remain debated [20,47].

Statins, prescribed primarily for cardiovascular risk reduction, have been associated in some observational studies with a lower risk of incident dementia, possibly through vascular and anti-inflammatory mechanisms, but findings are not uniform across cohorts [12,47]. Randomised trials and guideline appraisals have failed to demonstrate a clear, reproducible protective effect of lipid-lowering therapy on cognitive outcomes, and no statin currently carries an indication specifically for the prevention or treatment of cognitive impairment [12,20,47].

From a clinical standpoint, a multidimensional vascular risk management strategy—incorporating antihypertensive treatment, statin therapy where indicated, optimisation of glycaemic control, smoking cessation, and promotion of regular physical activity—is generally recommended for individuals with MCI, particularly when neuroimaging reveals small-vessel cerebrovascular disease [12,13,47,48].

#### 3.3.2. Deprescribing Anticholinergic and Sedative Medications

Cumulative exposure to drugs with strong anticholinergic properties is consistently associated with cognitive impairment and an increased risk of dementia. A large nested case–control study from the UK reported that higher cumulative doses of potent anticholinergics—including specific antidepressants, antipsychotics, bladder antimuscarinics, and antiparkinsonian agents—were linked to a significantly higher incidence of dementia in older adults [49]. Although observational data cannot establish causality, the dose–response gradient and biological plausibility have prompted guideline recommendations to minimise anticholinergic burden in individuals with, or at elevated risk of, cognitive impairment [18,19,20,21,49].

Long-term use of benzodiazepines and other sedative–hypnotics in older adults is associated with poorer cognitive performance, increased falls and functional decline, and has raised persistent concerns about a potential contribution to dementia, although confounding by indication remains difficult to fully disentangle [18,19,20,21,50]. In patients with MCI, benzodiazepines predictably impair attention and memory and heighten accident risk. Gradual tapering and substitution with non-pharmacological approaches to insomnia and anxiety—such as cognitive-behavioural therapy and structured sleep hygiene interventions—are therefore strongly encouraged [50].

Polypharmacy, often operationalised as the concurrent use of five or more medications, further amplifies the likelihood of drug–drug interactions and adverse cognitive effects [51]. Regular, systematic medication review—ideally using structured tools that quantify anticholinergic and sedative load—should be regarded as a core element of MCI management.

Iatrogenic hypoglycaemia and excessive blood pressure lowering represent additional, and sometimes overlooked, iatrogenic contributors. In older adults with diabetes, hypoglycaemic episodes have been associated with both acute delirium and subsequent chronic cognitive decline, supporting the need for individualised glycaemic targets that prioritise safety over tight control in people with MCI [52]. Similarly, overly intensive antihypertensive regimens may provoke symptomatic hypotension and cerebral hypoperfusion, potentially accelerating decline in frail, cognitively vulnerable patients [53].

### 3.4. Adjuvant and Nootropic Interventions

#### 3.4.1. *Ginkgo biloba* EGb 761^®^

Standardised *Ginkgo biloba* extract EGb 761^®^ has been studied for several decades in the treatment of cognitive impairment and dementia. Proposed mechanisms include antioxidant and anti-apoptotic effects, modulation of neurotransmission, improvement of cerebral microcirculation, and possible anti-amyloid actions [54]. A systematic review and meta-analysis of randomised, placebo-controlled trials in dementia and age-associated cognitive decline found that EGb 761^®^ (120–240 mg/day) produced modest but consistent improvements in cognition and global clinical impression compared with placebo, with small and somewhat variable effects on activities of daily living and a generally favourable tolerability profile [54]. By contrast, the Ginkgo Evaluation of Memory (GEM) trial, a large randomised study in older adults without dementia at baseline, showed that *Ginkgo biloba* 120 mg twice daily did not reduce the incidence of dementia over a median 6.1-year follow-up [55].

In the MCI spectrum, smaller randomised controlled trials and observational cohorts—including a multicentre Romanian study in amnestic MCI—have reported cognitive and functional benefits of EGb 761^®^, together with good overall tolerability [56]. On this basis, international expert and regional consensus statements assign EGb 761^®^ a class IIb recommendation, level of evidence A, as a symptomatic treatment option in MCI and early dementia [57,58]. EGb 761^®^ is not endorsed in major international guidelines (e.g., WHO, 2019) for routine dementia prevention or treatment, and use in MCI largely reflects regional practice patterns rather than global standards [18,19,20,21]. Clinical trials and meta-analyses have not demonstrated a clear overall excess of major bleeding events, but caution is warranted in patients receiving antiplatelet or anticoagulant therapy because of theoretical haemorrhagic risk [55].

#### 3.4.2. Citicoline (CDP-Choline) and Choline Alphoscerate

Citicoline and choline alphoscerate (αGPC) are cholinergic precursors that support phospholipid synthesis and may enhance cholinergic neurotransmission. Citicoline, or cytidine diphosphate–choline, is a product constituted by the combination of cytidine and choline, marketed as a food supplement [59,60]. αGPC is a choline-containing phospholipid precursor naturally present in the central nervous system (CNS) in small amounts and represents an intermediary stage in the degradation of phosphatidylcholine and a major choline donor [61].

Both compounds have been studied in vascular cognitive impairment, post-stroke cognitive decline, and MCI. A systematic review and meta-analysis (n = 7 studies) found that citicoline was associated with improvements in cognitive performance in MCI, Alzheimer’s disease, and post-stroke cognitive impairment, particularly in attention and executive functions, with a generally favourable tolerability profile [62]. However, many of the included trials were small, methodologically heterogeneous, and at risk of bias, which limits confidence in the conclusions.

Another systematic review and meta-analysis dedicated to αGPC in adult-onset cognitive dysfunctions (*n* = 7 RCTs and one prospective cohort study) showed significant effects for this compound in combination with donepezil on cognition, functional outcomes, and behavioral outcomes [63]. Studies included in this review were quite heterogeneous, with patients being diagnosed with various diseases, from AD to cerebral small vessel disease and depression, which limits the generalizability of the results.

More recently, a double-blind randomised controlled trial in 100 patients with amnestic MCI reported that αGPC 600 mg/day over 12 weeks led to improvements in ADAS-Cog scores compared with placebo, without serious adverse events [64]. Given the short follow-up and repeated cognitive testing, test–retest variability, including practice effects, should be considered when interpreting small score changes, particularly when functional endpoints and longer-term trajectories are not captured. This study provides some of the clearest direct evidence to date for a symptomatic benefit of a cholinergic precursor in MCI, but its short duration and modest sample size do not allow inferences about long-term trajectories or dementia prevention.

In day-to-day practice, citicoline and αGPC are sometimes used as adjunctive options in selected patients, particularly those with prominent vascular contributions, but they do not feature as standard, guideline-endorsed therapies for generic MCI in major international recommendations [18,19,20,21].

#### 3.4.3. Racetams

Racetam derivatives are grouped in three classes, according to their chemical structure, and these compounds have a long history as candidate cognitive enhancers [65,66]. For the topic of this review, the first class is of particular interest and includes piracetam, oxiracetam, aniracetam, pramiracetam, and phenylpiracetam, which were explored as add-ons for cognitive enhancement; oxiracetam and aniracetam are no longer in clinical use [66]. Early clinical trials in dementia and nonspecific cognitive impairment suggested modest benefits, but methodological weaknesses and inconsistent findings led to cautious conclusions in Cochrane reviews and subsequent summaries [67,68]. Contemporary, high-quality randomised controlled trials focused specifically on well-characterised MCI are essentially absent.

#### 3.4.4. Vitamins with Neuroprotective Potential

Vitamin B6, B12, and folate have attracted particular interest in MCI because of their role in homocysteine metabolism and potential effects on brain atrophy. In the VITACOG trial, 271 older adults with MCI were randomised to a combination of folic acid, vitamin B6, and vitamin B12 or placebo for two years [69]. Participants receiving B-vitamin supplementation showed a reduced rate of whole-brain atrophy and better performance on selected cognitive measures, with the greatest apparent benefit in those with elevated baseline homocysteine. Whether these structural and cognitive changes ultimately translate into a lower risk of progression to dementia remains unclear.

Vitamin E was assessed in the Alzheimer’s Disease Cooperative Study on MCI (ADCS-MCI) alongside donepezil. High-dose α-tocopherol (2000 IU/day) did not significantly decrease the rate of progression from amnestic MCI to AD over three years and did not provide a clinically meaningful cognitive benefit compared with placebo [27]. Given the absence of clear benefit and concerns raised in other populations about potential adverse effects of very high-dose vitamin E in older adults, these regimens lack support for routine use in MCI.

Low vitamin D levels have also been linked to cognitive impairment, and small randomised trials suggest that correcting deficiency may improve cognitive scores in older adults with MCI [70,71]. The available data, however, are heterogeneous and underpowered, and do not yet support firm, guideline-level recommendations.

#### 3.4.5. Neuropeptide-Based Interventions

Highly purified animal tissue extracts (HPATE), such as Cerebrolysin or Actovegin, are administered to patients with MCI, mNCDs, or MNCDs due to their neurotrophic properties, although their pharmacological properties are not well described [72]. Cerebrolysin is a porcine brain-derived neuropeptide preparation with putative neurotrophic and neuroprotective actions that has been studied for several decades in AD and vascular dementia [72,73]. Randomised and open-label trials in these populations suggest modest changes on cognitive measures and global impressions, with a generally favourable safety profile [74].

More recently, a small body of work has explored Cerebrolysin in pre-dementia stages, including amnestic MCI and first-degree relatives of patients with AD who themselves meet criteria for amnestic-type mild cognitive decline [74,75]. These studies report sustained improvements in memory and executive performance and, in some comparative analyses, lower observed rates of conversion to dementia in treated groups than in untreated comparators [74,75]. However, most originate from single-centre settings, involve limited sample sizes, and are characterised by suboptimal randomisation and blinding, with almost all data derived from Eastern European or Russian cohorts [74,75].

Actovegin is a biological product produced by ultrafiltration of calf blood, with the result being a deproteinated, pyrogen and antigen-free hemodialysate [72,76]. The ARTEMIDA trial explored the efficacy of Actovegin in post-stroke cognitive impairment during 12 months, in a parallel-group, andomised, multicenter, double-blind, placebo-controlled design (*N* = 248 participants) [77]. Actovegin had a favorable effect on cognitive outcomes (Alzheimer’s Disease Assessment Scale-cognitive subscale [ADAS-Cog]), and the compound was well tolerated [77]. An umbrella review investigating the efficacy and safety of post-stroke cognitive impairment therapies (*n* = 312 studies) concluded that Actovegin had a clinical effect significantly superior to placebo on the Montreal Cognitive Assessment (MoCA)-Cog scale, but little to no difference in neurological deficits or daily activities [78]. This dissociation between cognitive scale signals and functional endpoints limits the clinical interpretability of the observed score differences.

### 3.5. Neuropsychiatric Symptoms and Their Pharmacological Management

Neuropsychiatric, non-cognitive symptoms (NPSs)—including depression, anxiety, apathy, irritability, sleep disturbances, and, less frequently, psychotic phenomena—are highly prevalent in MCI [79,80]. Their presence is associated with poorer quality of life, increased caregiver burden, and, in several longitudinal studies, a higher likelihood of progression to dementia, most consistently for apathy [79,80]. Assessment should combine a semi-structured clinical interview with standardised rating instruments such as the Neuropsychiatric Inventory (NPI), complemented by validated depression and anxiety scales [81]. Systematic identification of reversible contributors (for example, pain, infection, sleep apnoea, polypharmacy, sensory deficits) is essential, as addressing these factors may improve both NPS and subjective cognitive complaints.

Management strategies place non-pharmacological approaches at the forefront: psychoeducation, cognitive-behavioural therapy, structured physical activity, sleep hygiene interventions, and support for social engagement represent first-line options [18,82]. When drug treatment is required, selective serotonin reuptake inhibitors (SSRIs) with low anticholinergic burden, such as sertraline or escitalopram, are generally preferred for clinically significant depression or anxiety, with attention to potential hyponatraemia and pharmacokinetic interactions [18,82,83,84]. Antidepressants with marked anticholinergic properties (for example, tricyclics or paroxetine) are generally discouraged in older adults with cognitive vulnerability because of anticholinergic burden and potential cognitive worsening [18,82,83,84]. Benzodiazepines should generally be minimised and, when used, restricted to brief courses, given their well-documented association with cognitive worsening and falls [50].

Antipsychotics are reserved for severe psychosis or agitation that endangers the patient or others, and should be prescribed at the lowest effective dose, for the shortest feasible duration, with explicit awareness of the increased risks of stroke and mortality in older adults [18,19,20,21,49,50,51,52,53]. These psychopharmacological measures are directed primarily at NPSs rather than core cognitive deficits, yet, when judiciously applied, they can markedly improve overall functioning and caregiver experience. They should always be combined with a structured review and reduction in medications that adversely affect cognition [18,19,20,21,49,50,51,52,53].

## 4. Discussion

The pharmacological management of MCI sits at an uneasy intersection between the intuitive desire to intervene early and the disappointing performance of most candidate drugs when tested rigorously in heterogeneous, mildly impaired populations. Classical Alzheimer’s treatments exemplify this tension. Cholinesterase inhibitors and memantine provide modest but clinically relevant benefits in established dementia, yet in MCI their effects are weaker, short-lived, and often difficult to distinguish from test–retest variability, including practice effects, especially when functional outcomes and conversion to dementia are considered [27,28,29,30,31,32,33,34,35,36,37]. In interpreting statistically significant changes on cognitive scales in MCI, we emphasise durability, concordance with functional endpoints, and overall risk–benefit over isolated short-term score changes. A consistent excess of adverse events further tips the risk–benefit balance against their routine use in this stage, a position explicitly reflected in current guideline recommendations [18,19,20,21]. A short, time-limited, and closely monitored trial of a cholinesterase inhibitor may be contemplated only in highly selected, progressive, biomarker-positive amnestic presentations, with predefined discontinuation criteria; even this strategy remains debated and is not endorsed as routine care in generic MCI. In the context of a sparse and outdated evidence base, and in the absence of endorsement from major guidelines, racetams cannot be recommended for the treatment of MCI. The reviewed findings argue for a selective, laboratory-guided approach for vitamin supplementation in MCI that should be directed toward documented deficiencies or markedly elevated homocysteine, rather than used as a routine, indiscriminate therapy. In the absence of robust data and formal endorsement from major international guidelines, Cerebrolysin and Actovegin cannot currently be regarded as standard therapy for MCI. Their use is better viewed as experimental or adjunctive, restricted to research protocols or to highly selected patients after explicit discussion of the limited and regionally concentrated evidence base. Where it is considered, EGb 761^®^ should be presented as an adjunctive option with modest expected benefit, and the same can be said about CDP-choline and αGPC in patients with MCI, based on the available evidence.

More recent strategies, particularly anti-amyloid monoclonal antibodies, represent a genuine attempt at disease modification in early AD. Even so, in carefully selected, biomarker-positive cohorts, the absolute gains in clinical scales remain modest and must be weighed against substantial treatment burden and non-trivial safety risks, including amyloid-related imaging abnormalities and the need for intensive monitoring [38,39,40,44,45,46,47]. At present, these agents are appropriate only for the relatively small subgroup of individuals with “MCI due to AD” who can be managed in specialised centres and who are willing to accept these trade-offs. There is no empirical or economic justification for their use in broader, biomarker-undefined MCI.

Neurotrophic and cholinergic precursors, herbal preparations, and vitamin-based approaches occupy an intermediate territory. They are biologically plausible, generally well tolerated, and, in some studies, associated with modest symptomatic gains, yet they lack convincing evidence for durable disease modification or a reliable reduction in conversion to dementia [54,55,56,57,58,62,64,67,68,69,70,71]. Signals of benefit tend to arise from small, single-region trials or from analyses at high risk of bias, and positive findings have often not been replicated across settings. Conceptually, their role is more coherent as adjuncts within a broader, multimodal strategy than as stand-alone, disease-modifying therapies.

A comparative overview of pharmacological options in MCI, including classical approaches for AD, disease-modifying antibodies, adjuvant agents, and indirect strategies, is presented in Table 1.

By contrast, the pharmacological interventions with the most consistent support in MCI are indirect rather than “pro-cognitive”: structured deprescribing of anticholinergic and sedative medications; rational optimisation of cardiovascular and metabolic regimens; and judicious treatment of neuropsychiatric symptoms. These measures are backed by convergent observational and interventional data, align closely with geriatric medicine principles, and carry a favourable balance of benefit and risk [18,19,20,21,47,48,49,50,51,52,53]. Their impact may be less dramatic than the promise of a “cognitive enhancer”, but they are more robustly anchored in real-world outcomes. Treatment of comorbid conditions that could alter the evolution of an MCI is strongly recommended, as the presence of such organic (e.g., neurologic, endocrine, autoimmune) diseases or psychiatric conditions (e.g., major depression, ADHD, anxiety disorders, chronic psychosis) can significantly alter the prognosis and case management of these patients [85,86,87,88,89]. According to the existing data, the evolution toward an MCI and the rate of conversion from MCI to dementia can be influenced by comorbid conditions [87,88,89].

An evidence-aligned, clinically coherent approach to MCI therefore requires resisting the reflex to medicalise every cognitive complaint with putative cognition-enhancing drugs. The real priority is accurate aetiological assessment, careful risk stratification, and thorough optimisation of general medical care. For this purpose, a critical role for the collaboration between primary care clinicians and AD specialists has been advocated by various authors [90,91,92]. Pharmacological interventions aimed directly at cognition should be used sparingly, in clearly circumscribed scenarios such as biomarker-confirmed “MCI due to AD” or borderline mild dementia, and always embedded within a comprehensive programme that emphasises non-pharmacological strategies, vascular risk modification, and systematic deprescribing.

### 4.1. Guideline-Level Synthesis and Practical Algorithm

On the basis of randomised controlled trials, meta-analyses, and contemporary guidelines, a pragmatic pharmacological approach to MCI can be framed in a series of simple steps. The main drug classes, together with their mechanisms, key evidence, and current guideline stance, are summarised in Table 1.

#### 4.1.1. Diagnosis Confirmation and Risk Stratification

The first priority is to confirm the diagnosis of MCI, distinguish it from both subjective cognitive decline and mNCDs, and establish an individual risk profile [93,94,95,96]. This includes determining the clinical subtype (amnestic versus non-amnestic, single- versus multi-domain), documenting biomarker status where available (amyloid, tau, neurodegeneration), interpreting detailed neuropsychological findings, and assessing vascular burden and the presence of neuropsychiatric symptoms (NPS) [7,9,10,11,12,13,44,45,79,80].

#### 4.1.2. Optimise Non-Pharmacological Management

Across phenotypes, non-pharmacological measures remain the foundation of care. Regular physical exercise, structured cognitive training, rigorous modification of vascular risk factors, targeted interventions for sleep and mood, and support for social engagement should be implemented systematically [12,13,18,19,20,44,45].

#### 4.1.3. Deprescribe and Optimise Existing Medications

A structured medication review is the next pharmacological pillar. This involves reducing anticholinergic and sedative load wherever possible, individualising antihypertensive, antidiabetic, and lipid-lowering regimens, and minimising overall polypharmacy [49,50,51,52,53]. Cumulative exposure to strong anticholinergics and long-term benzodiazepine use should be specifically targeted, given their association with cognitive impairment, falls, and functional decline [18,19,20,21,49,50]. Iatrogenic hypoglycaemia and excessive blood pressure lowering require particular attention in frail older adults with MCI [52,53].

#### 4.1.4. Use Adjunctive Symptomatic Agents Selectively

Against this background, adjunctive pharmacological agents may be considered in selected individuals, with the emphasis on modest symptomatic benefit rather than disease modification. Standardised *Ginkgo biloba* extract EGb 761^®^ can be used as a symptomatic option in regions where the evidence base and regulatory framework support its prescription, particularly in amnestic MCI, while acknowledging its limited effect size and potential bleeding concerns in patients on antithrombotic therapy [54,55,56,57,58]. Citicoline or choline alphoscerate (αGPC) may be added as cognitive adjuncts in patients with vascular or mixed profiles, although existing trials are relatively small and short-term [62,64]. Vitamin supplementation, such as B-vitamins or vitamin D, should be targeted strictly to those with documented deficiencies or markedly elevated homocysteine rather than prescribed empirically [27,69,70,71]. High-dose vitamin E is not supported for routine use in MCI, given the absence of clear benefit and potential safety concerns [27].

#### 4.1.5. Reserve Classical Alzheimer’s Drugs for Borderline or Clearly Demented Cases

Classical treatments used in Alzheimer’s disease—cholinesterase inhibitors (ChEIs) and memantine—should generally be reserved for situations in which the clinical picture is more compatible with mild dementia than with MCI. Exceptionally, a time-limited, carefully monitored trial may be contemplated in biomarker-positive, rapidly progressive amnestic MCI after explicit shared decision-making and with a predefined discontinuation strategy [18,19,20,21,27,28,29,30,31,32,33,34,35,36,37]. Routine prescription of ChEIs or memantine in unselected MCI populations is not supported by current evidence or guidelines.

#### 4.1.6. Restrict Anti-Amyloid Monoclonal Antibodies to “MCI Due to AD”

Anti-amyloid monoclonal antibodies (lecanemab, donanemab) should be restricted to biomarker-confirmed “MCI due to AD” or very mild AD managed in specialised centres, under rigorous safety monitoring and with clear communication about the modest absolute effect sizes, substantial treatment burden, and cost [38,39,40,44,45,46]. Their use in broader, biomarker-undefined MCI is neither evidence-based nor scalable.

Taken together, this framework underlines that, for most individuals with MCI, the dominant pharmacological opportunities are indirect: deprescribing, vascular and metabolic optimisation, and judicious treatment of neuropsychiatric symptoms. Agents targeting cognition directly should be deployed sparingly, in clearly circumscribed scenarios, and always embedded within a comprehensive non-pharmacological and psychosocial programme.

#### 4.1.7. Monitor the Evolution of Cognitive Outcomes

Clinical evaluation of MCI relies on a combination of brief screening instruments and, where available, formal neuropsychological testing. The Mini-Mental State Examination (MMSE) and the Montreal Cognitive Assessment (MoCA) remain the most widely used global cognitive screening tools [97,98,99]. The Quick Mild Cognitive Impairment screen (Qmci) and tablet-based composites such as the Brain Health Assessment, as well as automated neuropsychological batteries like the Cambridge Neuropsychological Test Automated Battery (CANTAB), enable more precise measurement of executive and processing-speed deficits that are poorly captured by global scales [99,100,101]. In routine practice, however, most guidelines still recommend the MMSE and MoCA as first-line tools, with more specialised instruments reserved for settings where adequate resources and locally validated norms are available.

### 4.2. Limitations of the Review and Future Perspectives of Research in This Field

This review is narrative in design and does not follow PRISMA methodology. Although we aimed to include the most relevant randomised controlled trials, meta-analyses, and guideline documents, some pertinent studies were probably missed. The restriction to English-language publications also introduces a risk of language and publication bias. In addition, the underlying literature is highly heterogeneous: MCI is defined in different ways, inclusion criteria vary, outcomes are measured with multiple scales, and follow-up periods are uneven. Under these conditions, formal cross-trial comparisons are fragile and effect sizes can only be interpreted in broad terms.

A substantial proportion of pharmacological studies in MCI were conducted before the widespread adoption of biomarker-based diagnostic frameworks and therefore enrolled participants with mixed underlying pathologies. By contrast, anti-amyloid therapy trials in early AD predominantly focus on biomarker-positive populations and may not fully represent community-dwelling individuals given a clinical label of MCI. Most available data also concern relatively fit older adults; evidence in the very old, in frail or multimorbid populations, and in non-AD MCI subtypes remains sparse. These gaps limit the generalisability of our conclusions and argue for a cautious, individualised application of the proposed recommendations.

Several recommendations for further investigations in the field of pharmacological treatment for patients with MCI refer to the need for clear, operationalized diagnostic criteria, as the prerequisite for large-scale, clinical trials dedicated to this pathology; necessary clarifications regarding the most appropriate instruments to monitor the neurocognitive deterioration in this population; and more epidemiological studies regarding the risk factors for MCI that could be mitigated through early therapeutic interventions or prophylactic strategies. Future research needs to prioritise large, biomarker-stratified randomised controlled trials that evaluate pharmacological agents within clearly defined MCI subtypes, together with pragmatic studies that integrate drug treatments, structured non-pharmacological interventions, and deprescribing protocols. Only through such designs can the true and proportionate role of pharmacology in MCI management be clarified.

## 5. Conclusions

Based on current evidence, no pharmacological agent can be recommended as standard therapy for unselected MCI. The traditional Alzheimer’s drug armamentarium—cholinesterase inhibitors (donepezil, rivastigmine, galantamine) and the NMDA receptor antagonist memantine—yields, at most, modest and short-lived improvements in cognitive test scores, without a durable reduction in progression to dementia and with a clear excess of adverse events. Anti-amyloid monoclonal antibodies represent the first plausibly disease-modifying option for biomarker-confirmed “MCI due to AD”, yet their clinical deployment should remain restricted to specialised centres and carefully chosen patients, given the combination of modest absolute effect sizes, considerable cost, and substantial safety and logistical demands.

Neurotrophic agents, cholinergic precursors, *Ginkgo biloba* extract, and vitamin-based strategies provide encouraging but not definitive signals of benefit and may be used as adjunctive options in selected cases—particularly where vascular contributions are prominent or where specific nutritional deficiencies or marked hyperhomocysteinaemia are documented. However, the most broadly applicable pharmacological leverage in MCI lies in systematic deprescribing and optimisation of concomitant therapies: reduction in anticholinergic and sedative load, avoidance of iatrogenic hypoglycaemia and excessive blood pressure lowering, and careful, individualised treatment of vascular risk factors.

## Figures and Tables

**Table 1:** Pharmacological options in mild cognitive impairment (MCI): mechanisms, main evidence, and current guideline stance.

Drug/Class	Primary Mechanism	Key Evidence in MCI/Very Early AD	Net Effect on Cognition/Progression	Guideline Stance in Generic MCI
Donepezil (ChEI)	↑ synaptic acetylcholine via AChE inhibition	ADCS-MCI (*N* = 769): donepezil vs. vitamin E vs. placebo, 3 year; 48-week RCT (*N* = 821); Cochrane + meta-analyses [27,28,29,30,31]	Small, transient cognitive gains; no durable effect on conversion; ↑ GI AEs and discontinuations	Not recommended routinely; at most short, monitored trial in highly selected, biomarker-positive, rapidly progressive amnestic MCI [18,19,20,21]
Rivastigmine (ChEI)	AChE and BuChE inhibition	InDDEx (≈1018 MCI, up to 4 year) [32]; small vascular CI studies [33]	No significant delay to AD; no clear global benefit; very high rate of mostly GI AEs	Not recommended for generic MCI
Galantamine (ChEI)	AChE inhibition + nicotinic modulation	GAL-INT (~1900 MCI) [34]; Cochrane review [35]	No relevant benefit on ADAS-Cog/CDR-SB or progression; ↑ GI AEs, high discontinuation	Not recommended in MCI; confined to dementia in guidelines
Memantine	NMDA receptor antagonism	Small pilot RCT in 25 aMCI [36]; reviews in mild AD [37]	Underpowered signal of benefit; overall, no convincing evidence in MCI, modest in mild AD	Not recommended in MCI; reserved for moderate–severe AD
Anti-amyloid mAbs (lecanemab, donanemab, aducanumab)	Amyloid-β clearance, disease-modifying intent	Lecanemab (CLARITY-AD) [38]; Donanemab (TRAILBLAZER-ALZ 2 and 4) [39,43]; Aducanumab EMERGE/ENGAGE, discordant, withdrawn [40]	~27–35% relative slowing of decline but small absolute CDR-SB differences; ARIA in a substantial minority; high burden and cost; no data in unselected MCI	Restricted to biomarker-confirmed “MCI due to AD”/very mild AD in specialised centres; not recommended for generic or biomarker-undefined MCI [18,19,20,21,38,39,40]
EGb 761^®^ (*Ginkgo biloba*)	Antioxidant, microcirculatory, and possible anti-amyloid	RCTs/meta-analyses in dementia/age-related CI [54]; GEM negative for prevention [55]; smaller MCI studies include Romanian amnestic MCI [56]	modest symptomatic gains in cognition and, variably, ADL; no prevention of dementia; good tolerability, theoretical bleeding concern on antithrombotics	Optional adjunct in some regional consensus (IIb/A) [57,58]; not endorsed by WHO/NICE for routine MCI [18,19,20,21]
Citicoline (CDP-choline)	Cholinergic precursor; membrane phospholipids	Systematic review/meta-analysis in MCI/AD/post-stroke [62]	Signals of improved attention/executive function; trials small, heterogeneous, at risk of bias	Adjunctive option in selected vascular/mixed MCI; not guideline-standard
Choline alphoscerate (αGPC)	Cholinergic precursor; phosphatidylcholine	RCT in 100 aMCI, 12 weeks [64]	Improved ADAS-Cog short term; no long-term/progression data; good tolerability	Adjunctive in selected cases; not guideline-standard
Racetams (e.g., piracetam)	Membrane/neurotransmission modulation (unclear in vivo)	Older trials in dementia/nonspecific CI; Cochrane review [67,68]; no modern MCI RCTs	Outdated, inconsistent evidence; no robust contemporary MCI data	Not recommended; no guideline support
Vitamin B6/B12/folate	Homocysteine lowering; neuroprotective plausibility	VITACOG (*N* = 271 MCI) B-vitamin vs. placebo, 2 year [69]	↓ brain atrophy and better some cognitive measures, especially with high homocysteine; the effect on conversion is unclear	Targeted use only in documented deficiency/high homocysteine; not for routine supplementation
Vitamin E (high-dose)	Antioxidant	ADCS-MCI high-dose α-tocopherol 2000 IU/day [27]	No reduction in conversion; no clear cognitive benefit; safety concerns at very high doses	Not recommended for MCI [27]
Vitamin D	Neurosteroid, immune/vascular effects	Small, heterogeneous trials in deficient older adults with MCI [70,71]	Possible cognitive improvement when correcting deficiency; evidence underpowered	Correct deficiency, but no support for supra-physiologic or routine MCI supplementation
Cerebrolysin, Actovegin	Neuropeptide mixture, neurotrophic/neuroprotective	RCTs/open-label in AD/vascular dementia [74]; small single-centre MCI/at-risk relative studies [74,75]; ARTEMIDA trial [77]	Suggestive cognitive and progression benefits in selected cohorts; limited by small, regional, methodologically weak studies	Experimental/adjunctive only; not guideline-endorsed, restricted to research/highly selected patients
Indirect strategies: deprescribing, vascular/ metabolic optimisation	Reduce drug-induced cognitive toxicity; improve cerebrovascular/metabolic milieu	Anticholinergics [49]; benzodiazepines [50]; polypharmacy [51]; hypoglycaemia [52]; hypotension [53]; vascular risks [47,48]; guideline support [18,19,20,21,44,45]	Strong convergent support: ↓ anticholinergic/sedative burden, avoid extremes of BP/glucose, treat vascular risks → better or more stable cognition, fewer adverse events	Core component of MCI management; recommended as priority intervention in guidelines [18,19,20,21,44,45]

AD = Alzheimer’s Dementia; ADCS = Alzheimer’s Disease Cooperative Study; ChEI = cholinesterase inhibitors; AChE = acetylcholinesterase; BuChE = butyrylcholinesterase; AE = adverse effects; ARIA = Amyloid-related imaging abnormalities; GEM = Ginkgo Evaluation of Memory; GI = gastrointestinal; MCI = Mild cognitive impairment; RCT = randomized clinical trial; ↑ = increased; ↓ = decreased.

## Data Availability

No new data were created in this study. Data sharing is not applicable to this article.

## Abbreviations

The following abbreviations are used in this manuscript:

AChE	acetylcholinesterase
AD	Alzheimer’s Dementia
ADAS-Cog	Alzheimer’s Disease Assessment Scale–Cognitive Subscale
ADCS	Alzheimer’s Disease Cooperative Study
AE	adverse effects
aMCI-SD	amnestic single-domain
aMCI-MD	amnestic multi-domain ARIA
ARIA	Amyloid-related imaging abnormalities
CDR-SB	Clinical Dementia Rating–Sum of Boxes
ChEI	cholinesterase inhibitors
DSM	Diagnostic and Statistical Manual of Mental Disorders
ICD	International Classification of Diseases
GEM	Ginkgo Evaluation of Memory
GI	gastrointestinal
MCI	Mild cognitive impairment
mNCD	mild neurocognitive disorder
MNCD	major neurocognitive disorder
naMCI-SD	non-amnestic single-domain
naMCI-MD	non-amnestic multi-domain
RCT	randomized clinical trial
